# Reclaiming time as a fundamental determinant of health equity

**DOI:** 10.1016/j.ssmph.2026.101939

**Published:** 2026-06-22

**Authors:** Berta Valente

**Affiliations:** a EPIUnit ITR, Instituto de Saúde Pública da Universidade do Porto, Universidade do Porto, Porto, Portugal; b Departamento de Saúde Pública e Ciências Forenses e Educação Médica, Faculdade de Medicina, Universidade do Porto, Alameda Prof. Hernâni Monteiro, 4200-319, Porto, Portugal; c Health Economics Unit, Department of Clinical Sciences (Malmö), Faculty of Medicine, Lund University, 221 00, Lund, Sweden

**Keywords:** Time poverty, Temporal inequality, Social determinants of health, Health equity, Epidemiology, Care work

## Abstract

Health is widely recognized as shaped by social and structural conditions, yet time remains largely absent from dominant public health frameworks. This paper brings together sociological and epidemiological perspectives to argue that time is a fundamental dimension of health that has been insufficiently articulated. Rather than proposing a new determinant, it reframes time as an essential condition through which social inequalities are produced and experienced. In public health research, time is most often treated as a technical parameter, such as duration or life course stage, obscuring how it is socially organised and unequally distributed. Drawing on interdisciplinary scholarship, the present paper highlights two interrelated dimensions of time - availability and intensity - and shows how temporal scarcity, acceleration, and uncertainty are patterned by social position, gender, and economic insecurity. It examines how constrained time shapes everyday possibilities for care, rest, and participation, while ill health and caregiving further restrict control over time, reinforcing cycles of disadvantage. The paper also reflects on how healthcare systems are shaped by standardised clock time, often misaligning with the lived realities of chronic illness and long-term care. By synthesising existing scholarship and making time visible as a condition shaping agency, dignity, and health, this paper seeks to strengthen critical public health debates and encourage greater attention to temporal inequalities in research, policy, and practice.

## Highlights

• Time operates as health determinant through availability and intensity dimensions.
• Temporal inequality patterns by gender, income, and economic precarity.
• Bidirectional cycles: time scarcity affects health; illness restricts time.
• Epidemiological models should treat time as structural exposure, not parameter.
• Policy implications span labor regulations, healthcare delivery, and care work.

## 1 Time, health and social structure

The definition of health has evolved and is currently understood as a “state of complete physical, mental and social well-being and not merely the absence of disease or infirmity” (Basic Documents, 2020). In response, numerous frameworks have been developed to address the social determinants of health and to advance health equity (Carmichael et al., 2023). Yet one fundamental dimension remains largely absent from these approaches: time (Strazdins et al., 2016). This paper argues that time should be explicitly recognized as a socially structured determinant of health, shaping agency, dignity, and health inequalities.

## 2 The limits of neutral time in epidemiology

Within epidemiology, time is most often treated as a technical and quantitative variable. It appears as the timing of disease onset, the duration of exposure, the number of years lived with ill health (e.g. disability-adjusted life years), or as sensitive or critical periods of vulnerability (Porta et al., 2014). Time is also implicitly understood as a resource required to adopt health-promoting behaviors, access healthcare services, sustain social relationships or generate income (Harrison et al., 2024). These perspectives largely reduce time to a neutral container, neglecting how time itself is socially produced, unequally distributed, and differently experienced (Šubrt, 2021).

Sociological perspectives offer a broader understanding of time that is highly relevant for public health. Time is not only measured through clocks and calendars, but also lived and experienced as duration, pace, and anticipation, shaped by social institutions, economic relations, and the finite trajectory of human life (Šubrt, 2021). Importantly, perceptions of time are closely linked to individuals’ sense of identity, agency, and control over their lives (Strazdins et al., 2016). What people mean when they report “lacking time” therefore cannot be understood just in terms of insufficient hours, but must also account for how time is structured, valued, and constrained within social contexts (Strazdins et al., 2016).

## 3 Lived temporal constraints, care, and health inequalities

Time operates as a determinant of health through two interrelated dimensions: its availability and its intensity. Beyond the absolute quantity of time, the experience of rushing, acceleration, and constant pressure also shapes everyday life (Rosa & Trejo-Mathys, 2015). In many contemporary market-driven societies, strong emphases on productivity and efficiency have accelerated daily rhythms, intensifying temporal demands at work and at home (Kalenkoski et al., 2011; Rosa & Trejo-Mathys, 2015; Wajcman, 2008). Technological change, urbanization, and flexible labor markets have contributed to “boundaryless” forms of work and an increased pace of activity, generating time-related stress and reinforcing the persistent devaluation of care (Albertsen et al., 2010; Rosa & Trejo-Mathys, 2015; Wajcman, 2008). Within the labour market, temporal pressures are mainly organized through two interrelated dimensions of working time: time control and time variability. Working time control refers to employees’ autonomy over how, when, and where they work, shaping their capacity to protect time for rest, care, and other non-market activities (Backhaus, 2022). Working time variability captures the extent to which working hours fluctuate over time, reflecting the stability of temporal rhythms and thus the lived intensity and predictability of work (Backhaus, 2022). Both dimensions vary across Europe, working time regimes and have implications for health: higher working time variability, especially when combined with low working time control, is associated with worse self-rated health, more psychosomatic complaints, and more frequent sleep problems (Artazcoz et al., 2024; Backhaus, 2022). Furthermore, multiple time-related indicators, such as discretionary time, subjective time scarcity, and objective measures of time poverty and time excess might mediate the association between socioeconomic status (SES) and health, since the health implications of “time poverty” differ for high- and low-SES groups (Bó, 2022).

Individuals working multiple jobs and those combining paid employment with caregiving responsibilities experience particularly severe constraints on both time availability and temporal intensity (Kalenkoski et al., 2011; Šubrt, 2021). Women are overrepresented in these situations and, globally, spend on average 2.8 more hours per day than men on unpaid care and domestic work (Hanna et al., 2023). Given that women, especially those living with lower incomes, continue to perform the largest share of unpaid domestic and care work, inequalities in time constitute an important mechanism through which social and gender disparities in health are reproduced (Bó, 2022; Wajcman, 2008). These inequalities are further deepened in contexts of migration, armed conflict, and economic instability, where uncertainty, disruption, and chronic temporal strain become defining features of everyday life, with negative consequences for health and well-being.

Time and health are linked through a bidirectional relationship that reinforces disadvantages. Constraints on time availability and intensity limit engagement in health-promoting behaviors, such as physical activity or preparing nutritious meals, while fostering fatigue, negative mood, and stress-related physiological responses (Albertsen et al., 2010; Artazcoz et al., 2024; Kalenkoski et al., 2011).

At the same time, living with illness or disability reshapes how people can use and control their time, restricting participation in paid work, caregiving, and social life (Albertsen et al., 2010). Treatment burden research shows that chronic illness management often entails substantial work outside formal care, such as information seeking, medication management, appointments, self-monitoring, lifestyle changes, financial tasks, and system navigation (Eton et al., 2020). Treatment burdens are also socially patterned, since most treatment measures were developed in high-income settings and may not adequately reflect the experiences of groups with lower health literacy, limited access to care, or greater socioeconomic constraints (Mendoza-Quispe et al., 2023). These dynamics contribute to socially patterned cycles in which poor health and constrained time mutually reinforce inequalities in mental and physical health (Strazdins et al., 2016).

## 4 Reframing time for health equity

These unequal temporal conditions are not only experienced in everyday life but are also embedded in the organisation of healthcare systems, which are deeply structured by temporal assumptions. Care is organized around clock time, standardized schedules, and linear treatment trajectories, often failing to capture the lived temporalities of chronic illness, disability, or long-term care (Albertsen et al., 2010). These misalignments disproportionately affect people in socially disadvantaged positions, including those balancing paid work and caregiving or living with limited material resources. People living with illness and caregivers must navigate overlapping temporal scales, trade present time for uncertain future benefits, and adapt to care processes that rarely align with everyday rhythms (Albertsen et al., 2010). Attending to these multiple temporalities reveals whose time is recognized and resourced within health systems and highlights the social and political dimensions of temporal organization in care delivery.

As epidemiologists and public health professionals working in an increasingly complex social and political landscape, and committed to health as a fundamental human right, we must make time visible as a determinant of health. Epidemiological models often treat time as neutral or exogenous, obscuring how temporal scarcity, acceleration, and uncertainty function as socially patterned exposures. Making these dimensions explicit in causal frameworks reveals how time structures mediation, accumulation, and constraint across the life course, and helps explain why conventional analyses may systematically misattribute responsibility to individual behaviour in ways that reproduce inequities.

Addressing this limitation requires conceptualizing time not only as a methodological parameter, but as a structural exposure that can be measured and incorporated into epidemiological models. Attention to temporal availability and intensity has direct implications for study design, measurement strategies, and the interpretation of social inequalities in health. Existing European and international time-use infrastructures, such as the Harmonised European Time Use Surveys and the Multinational Time Use Study, already operationalize these temporal dimensions by providing harmonised 24-h diary data on paid work, unpaid care and domestic work, personal care (including sleep), travel, social and leisure activities, together with detailed sociodemographic information, thereby enabling the analysis of temporal availability and intensity across social groups and countries (Centre for Time Use Research, 2020; EUROSTAT, 2020)**.** Systematically including temporal dimensions alongside sociodemographic characteristics allows epidemiological research to better reflect the lived experiences of different population groups and to recognize time as a condition shaping agency, dignity, and health, rather than merely a resource for individual behaviour change.

At the primordial prevention level (Porta et al., 2014) this perspective calls for engagement through public health diplomacy (Joshi et al., 2025). Researchers and practitioners can contribute by advocating health in all policies, supporting policymaking beyond the health sector that protects time as a social good. This includes engagement with several sectors such as with social protection systems that buffer economic uncertainty, labour policies that regulate working hours and job security, housing policies that shape residential stability and commuting time or transport infrastructures that reduce time poverty and temporal strain. Equally important is the inclusion of family-related policies in national agendas, such as paid parental leave, accessible childcare, and support for elder care (Jaggi & Gupta, 2025), which can ease the temporal pressures on working-age adults who provide care across generations. Within healthcare systems themselves, aligning institutional time with patients’ lived temporal rhythms may involve models of care that coordinate multiple appointments or services within a single visit, thereby reducing the time costs and treatment burden associated with fragmented care (Bell et al., 2024). Such efforts advance health equity by ensuring that people have the temporal conditions necessary to exercise choice, participate in society, and live healthy lives. Reclaiming and framing time as a determinant in public health practice is fundamental to advancing health equity and population well-being.

## Ethics statement

Not Applicable.

## Funding

This work was supported by FCT - Fundação para a Ciência e Tecnologia, I.P. through the projects with references UID/4750/2025 and LA/P/0064/2020 and DOI identifiers https://doi.org/10.54499/UID/04750/2025 and https://doi.org/10.54499/LA/P/0064/2020, and the doctorate scholarship with reference 2023.00992. BD and DOI identifier https://doi.org/10.54499/2023.00992.BD.

## Competing interests

None to declare.

## Data Availability

No data was used for the research described in the article.
